# Thermodynamics of Mg–Al Order-Disorder Reaction in MgAl_2_O_4_-Spinel: Constrained by Prolonged Annealing Experiments at 773–1123 K

**DOI:** 10.3390/molecules26040872

**Published:** 2021-02-07

**Authors:** Yunlu Ma, Xinjian Bao, Xi Liu

**Affiliations:** 1 School of Earth and Space Sciences, Peking University, Beijing 100871, China; Yunlu.Ma@pku.edu.cn (Y.M.); Xinjian.Bao@pku.edu.cn (X.B.); 2 Key Laboratory of Orogenic Belts and Crustal Evolution, Ministry of Education of China, Beijing 100871, China

**Keywords:** cation exchange equilibrium, geothermometer, low-*T* annealing, MgAl_2_O_4_-spinel, single-crystal X-ray diffraction, thermodynamic model

## Abstract

MgAl_2_O_4_-spinel has wide industrial and geological applications due to its special structural and physical–chemical features. It is presumably the most important endmember of complex natural spinel solid solutions, and therefore provides a structural model for a large group of minerals with the spinel structure. There exists a well known but still inadequately understood phenomenon in the structure of MgAl_2_O_4_-spinel, the Mg–Al cations readily exchanging their positions in response to variations of temperature, pressure, and composition. A large number of experiments were performed to investigate the Mg–Al cation order-disorder process usually quantified by the inversion parameter *x* (representing either the molar fraction of Al on the tetrahedral T-sites or the molar fraction of Mg on the octahedral M-sites in the spinel structure), and some thermodynamic models were thereby constructed to describe the *x*-*T* relation. However, experimental data at some key *T* were absent, so that the different performance of these thermodynamic models could not be carefully evaluated. This limited the interpolation and extrapolation of the thermodynamic models. By performing some prolonged annealing experiments with some almost pure natural MgAl_2_O_4_-spinel plates and quantifying the *x* values with single-crystal X-ray diffraction technique, we obtained some critical equilibrium *x* values at *T* down to 773 K. These new *x*-*T* data, along with those relatively reliable *x* values at relatively high *T* from early studies, clearly indicate that the CS94 Model (a model constructed by Carpenter and Salje in 1994) better describes the Mg–Al cation order-disorder reaction in MgAl_2_O_4_-spinel for a wide range of *T*. On the basis of the CS94 Model, a geothermometer was established, and its form is *T*_-closure_ = 21362 × *x*^3^ − 12143 × *x*^2^ + 6401 × *x* − 10 (*T*_-closure_ standing for the closure temperature of the Mg–Al cation exchange reaction). This geothermometer can be used to constrain the thermal history of the geological bodies containing MgAl_2_O_4_-spinel.

## 1. Introduction

Due to its special physical and thermal properties, such as high melting point (2408 K), good mechanical strength (135–216 MPa at room temperature and 120–205 MPa at 1573 K), and low thermal expansion coefficient (~9 × 10^−6^ K^−1^ between 303 and 1673 K; [1,2,3]), MgAl_2_O_4_-spinel has many industry applications and is commonly used as refractories, catalysts, and humidity sensors, etc. [4,5,6,7,8,9]. In the field of Earth sciences, MgAl_2_O_4_-spinel frequently occurs in the crust and shallow mantle and sets its influence on the physical, chemical, and dynamical evolution of the Earth [10,11]. Moreover, it is one of the most important endmembers of complicated natural spinel solid solutions and provides a fundamental structural model for many minerals with the spinel structure [12,13,14,15]. Hence, any study on the structural features of MgAl_2_O_4_-spinel may have important applications to the spinel-structured minerals such as magnetite, chromite, and ringwoodite [16,17,18,19].

MgAl_2_O_4_-spinel is cubic and has the space group *Fd*$\bar{3}$*m*. Its O atoms closely pack and form an approximately cubic array, in which two types of cavities—tetrahedral T-sites and octahedral M-sites—occur. Its Mg cations are usually assumed to occupy the T-sites only and its Al cations to occupy the M-sites only, resulting in a normal spinel configuration. Due to variations of temperature (*T*), pressure (*P*), and composition, however, it is well known that some Al and Mg cations may exchange their positions, as described by Equation (1) [20,21,22,23,24,25]:

^T^Al + ^M^Mg ⇌ ^T^Mg + ^M^Al (1)

The backward reaction of this equation, i.e., the disordering process, refers to the process in which some Al cations on the M-sites relocate onto the T-sites and some Mg cations on the T-sites relocate onto the M-sites. Vice versa, the forward reaction, i.e., the ordering process, represents the process in which some Al cations on the T-sites return to the M-sites and some Mg cation on the M-sites return to the T-sites. The actual structural formula of MgAl_2_O_4_-spinel is then better expressed as

^[4]^(Mg_1−*x*_Al*_x_*)^[6]^(Mg*_x_*Al_2−*x*_)O_4_ (2)

in which *x* is the inversion parameter varying from 0 to 1, indicating the atomic fraction of Al on the T-site or Mg on the M-site, ^[4]^ refers to the T-sites, and ^[6]^ refers to the M-sites. When *x* = 0, *x* = 2/3, and *x* = 1, the Mg–Al cations in the structure respectively attain a normal cation distribution, a completely disordered cation distribution, and an inverse cation distribution, and the spinels are respectively called as a normal spinel, a fully random spinel, and an inverse spinel. As the *x* value of MgAl_2_O_4_-spinel increases, the average bond lengths of the T-O bonds (*d*_T-O_) and M-O bonds (*d*_M-O_) respectively decrease and increase [26,27], and the unit-cell parameter (*a*), the oxygen coordinate parameter (*u*), and the unit-cell volume (*V*) all reduce [27]. These micro structural changes have important influences on some macro physical–chemical properties, such as thermal expansion, electrical conductivity, vibrational features, elasticity, trace element partition, etc. [26,28,29,30,31].

The Mg–Al cation order-disorder process also influences the Gibbs free energy (G), configurational entropy ($S_{C}$), and non-configurational entropy ($S_{D}$), internal energy (U), and enthalpy (H), and so on [32,33,34,35,36]. Its energetics can be described by the following equation,

(3) $$\Delta G_{D}=\Delta U_{D}-T\left(\Delta S_{C}+\Delta S_{D}\right)+P\Delta V_{D}$$

where $\Delta V_{D}$ refers to the change of volume caused by the Mg–Al cation exchange reaction, etc. Based on this equation, some thermodynamic models have been established to describe the relationship between *x* and *T*, and the best known three are

(4) $$-\frac{\Delta H_{D,\;\mathrm{int}}}{RT}=\ln\frac{x^{2}}{\left(1-x\right)\left(2-x\right)}$$

(5) $$-RTln\left(\frac{x^{2}}{\left(1-x\right)\left(2-x\right)}\right)=\alpha +2\beta x$$

and

(6) $$T=T_{C}+\frac{T_{C}}{\left(c^{\prime }-1\right)Q}\left(1-c^{\prime }Q^{5}\right)$$

[32,33,36,37] (the Q variable in Equation (6) is called as the order parameter, which equals 1–1.5*x*). To accurately determine the parameters in these thermodynamic models (Equation (4), hereafter briefed as the NK67 Model; Equation (5), hereafter briefed as the ON83 Model; Equation (6), hereafter briefed as the CS94 Model), a wide range of equilibrium *x* values for a wide range of *T* values is critical. Consequently, a large number of experimental studies at different *T* and 1 atm have been carried out to investigate the Mg–Al cation exchange process, with the experimental *T* varying from 873(5) to 1887(10) K and the inversion parameter *x* varying from 0.146(15) to 0.390(39) [23,27,38,39,40,41,42].

There are, however, some general problems in these experimental results. For the in situ experiments at relatively high *T* (e.g., *T* > 1573 K) where the cation order-disorder reaction is quick, either the accuracy of the *T* measurements is generally low due to the employment of inappropriate thermocouple [40], or the uncertainty of the *x* values is too large because of the quantifying method used [41]. For the experiments performed at high *T* with conventional quench method, the *x* values might be significantly modified by the quench process due to high Mg–Al cation exchange reaction rates [38,42,43]. For the experiments conducted at relatively low *T* (e.g., *T* < 973 K), the Mg–Al cation exchange proceeds extremely slowly and a very long experimental time is needed in order to reach close equilibrium [42]. For example, the experiment at 873 K conducted by Andreozzi et al. [27] ran for 45 days. As a result, accurate and reliable *x*-*T* data for the Mg–Al cation exchange process in MgAl_2_O_4_-spinel are still somewhat limited and cannot tell the difference in those thermodynamic models [43].

To extend the *x*-*T* data range and thus obtain better constraints for the Mg–Al cation exchange thermodynamic models, we performed some experiments at relatively low *T* (773–1123 K) with very long heating durations (from 24 h to 360 days). We used a nearly pure natural MgAl_2_O_4_-spinel crystal as the starting material. To ensure close equilibrium in these experiments, the heating durations were set to at least twice the required heating durations for close cation ordering-disordering equilibrium, as implied by the kinetics study in the literature [42,43]. After quenching, our samples were analyzed by using the single-crystal X-ray diffraction method. These new *x*-*T* data were added together with those reliable *x*-*T* data recently assembled by Ma and Liu [43] to obtain new parameters for the Mg–Al cation exchange thermodynamic models. 

## 2. Experimental and Analytical Methods

### 2.1. Natural MgAl_2_O_4_-Spinel Crystal

A natural gem-quality spinel crystal (SP3) was purchased from Mogok (Burma) and used in our experiments. It was red, 2~3 mm in size, and of perfect octahedral shape. Under optical microscope, it was almost transparent and clear, with only a few tiny unknown fluid/solid inclusions. 

The composition of this crystal was determined as Mg_1_._000(3)_Fe_0_._006(1)_Ti_0_._002(1)_Al_1_._979(3)_Cr_0_._011(1)_O_4_ by using a JEOL JXA-8230 electron microprobe (EMP) in wavelength dispersive mode (10 EMP analyses obtained with a 15 kV accelerating voltage, a 10 nA beam current, and a 2 μm beam spot). According to Ma and Liu [43], these low levels of Fe, Ti, and Cr cations should have negligible influences on the Mg–Al cation exchange reaction. We thus ignored these trace impurities in this study and treated the composition of this natural spinel as MgAl_2_O_4_.

This crystal and the sample N-Sp used in Liu et al. [25] were from the same batch of sample-purchasing and attained similar initial *x* values (*x*_-int_ = ~0.145; Table 1), as determined by single-crystal X-ray diffraction analysis (to be reported somewhere else). Nevertheless, the initial *x* value of our natural spinel is not important to this study.

The SP3 crystal was cut along its (111) crystal planes into five small plates by using a low-speed diamond saw.

### 2.2. Annealing Experiments from 773 to 1123 K

Five annealing experiments were conducted from 773 to 1123 K and at room *P* (Table 1). Two KSL-1100X muffle furnaces were used in these experiments. Al_2_O_3_ crucibles were used to hold the samples and were placed near the hot junction of the controlling thermocouple (K-type) of the furnaces. The accuracy of the experiment *T* should be ~ ±5 K. The heating durations of these experiments were estimated to ensure close cation-exchange equilibrium according to Ma and Liu [43] (*t_-cal_* in Table 1). To account for potential uncertainty in extrapolating the kinetics model down to low *T*, the real heating durations (*t_-exp_* in Table 1) were set significantly longer than the estimates (i.e., *t_-cal_*). We therefore believe that the cation exchange reaction in our experiments should have closely approached its equilibrium. At the end of an annealing experiment, the sample, along with the crucible, was quickly removed from the furnace and immediately quenched in cold water (less than 1 min for the whole process), which should not have had any influence on the *x* value of the sample attained at high *T* [43].

All the MgAl_2_O_4_-spinel plates were first heated at 1123 K for 24 h (Run HT3-1; Table 1), with one sample plate randomly selected and defined as the experimental product of this experiment (SP3-1), with another sample further annealed at 1023 K for 7 days (Run HT3-2) and defined as sample SP3-2, and with another sample further annealed at 923 K for 30 days (Run HT3-3) and defined as sample SP3-3. The remaining two MgAl_2_O_4_-spinel plates from Run HT3-1 were heated at 823 K for 120 days (Run HT3-4; Table 1), with one sample plate randomly selected and defined as the experimental product of this experiment (SP3-4) and with the other sample further annealed at 773 K for 360 days (Run HT3-5) and defined as sample SP3-5.

### 2.3. Analyzing Methods

Single-crystal X-ray diffraction data of these annealed MgAl_2_O_4_-spinel sample plates were collected from 4.37 to 31.32° using an XtaLAB Synergy-R micro-focused four-circle diffractometer in the Analytical Instrumentation Center of Peking University (Mo Kα radiation with λ = 0.71073 Å) or from 4.37 to 28.30° using an XtaLAB AFC12 micro-focused four-circle diffractometer in the College of Engineering, Peking University (Mo Kα radiation with λ = 0.71073 Å). These data were processed by using the SHELXT software included in the SHELXTL package.

First, the initial structure solutions of these samples were obtained by using the direct method. Then, under the chemical constraint determined by the EMP analyses, the initial structure was refined with the full-matrix least-squares method. Relying on the Fourier electron density maps, the coordinates of the Mg and Al atoms were located unequivocally. Further, using the difference electron density maps, the O atoms were subsequently found. Finally, the anisotropic displacement parameters of all atoms were refined.

The *x* values directly obtained from the above structural refinements would bear large uncertainty due to the reason that the Mg and Al cations have similar X-ray scattering factors. To acquire more accurate *x* values, the bond-length method [20] (Equation (7)) was adopted,

(7) $$F\left(X_{i}\right)=\sum_{j}{\left\{\left[O_{j}-C_{j}\left(X_{i}\right)/\sigma_{j}\right]\right\}}^{2}$$

In this equation, the $O_{j}$ stands for the observed quantity, including the *d*_T-O_ and *d*_M-O_, the mean atomic numbers of the T-sites and M-sites, the number of charges for charge balance, the atomic proportions obtained from the EMP analyses, and so on, and the $\sigma_{j}$ represents the corresponding standard deviation. The $C_{j}\left(X_{i}\right)$ refers to the corresponding quantity for the $O_{j}$ calculated by variable cation fractions $X_{i}$. To derive the *x* value, multiple cycles of minimizing the $F\left(X_{i}\right)$ values were executed, and the minimizing process stopped when the difference between the calculated and observed quantity was less than 1δ [25]. This equation simultaneously took the structural and chemical data into account.

We used the ionic radius values determined in Lavina et al. [44] in minimizing the $F\left(X_{i}\right)$ value. In addition, the following assumptions were applied according to Carbonin et al. [20] and relevant cation exchange studies [32,45,46]: (a) Mg, Al, Fe^2+^, Fe^3+^, and vacancies can fill in both the T-sites and M-sites; (b) Cr and Ti fill in the M-sites only; (c) Bond lengths are a linear combination of the site atomic fractions multiplied by their characteristic bond distances in the 2-3 spinels.

## 3. Results and Discussions

After being annealed at different temperatures for different amounts of time, our MgAl_2_O_4_-spinel sample plates showed no change in color, transparency, or composition.

The key parameters of our annealing experiments and results of our structural refinements are summarized in Table 1. The details of the structure refinements are shown in the Supplementary Materials (Tables S1–S5). The Cif files are also shown as Supplementary Materials (Cif HT3-1 to CifHT3-5).

Although no reversal experiment was carried out in this study, it is believed that our annealing experiments in the *T* range of 773–1123 K should have reached close Mg–Al cation exchange equilibrium. Firstly, the heating duration required for good cation exchange equilibrium in every experiment was estimated according to previous kinetics study [42,43]. To secure good equilibrium, secondly, the real heating durations in our experiments were at least 2 times the estimates (Table 1). Thirdly, our results and those reversed results obtained by Andreozzi et al. [27] at the same *T* are virtually identical, as revealed in our later discussion. 

**Figure 1:**
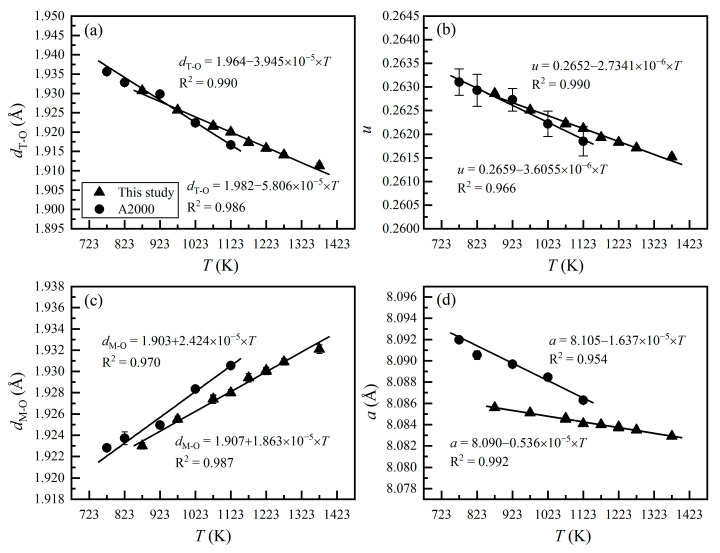
Correlations between temperature (*T*) and crystal structure parameters of MgAl_2_O_4_-spinel: (**a**) *d*_T-O_ vs. *T*; (**b**) *u* vs. *T*; (**c**) *d*_M-O_ vs. *T*; (**d**) *a* vs. *T*. A2000, Andreozzi et al. [27]. Note that synthetic stoichiometric MgAl_2_O_4_-spinels were used in Andreozzi et al. [27].

Figure 1 shows the crystal structural parameters of our MgAl_2_O_4_-spinel samples annealed at different *T*. Similar to the single-crystal X-ray refinement results from Andreozzi et al. [27], our data define good linear correlations between these parameters and *T*. In detail, *d*_T-O_ decreases from 1.93562(5) to 1.91666(1) Å (Figure 1a), *d*_M-O_ increases from 1.92281(8) to 1.93054(3) Å (Figure 1c), *u* decreases from 0.2631(3) to 0.2619(3) (Figure 1b) and *a* decreases from 8.0920(3) to 8.0863(1) Å (Figure 1d), as *T* increases from 773 to 1123 K.

Good agreement can be observed between the results of this study and those from Andreozzi et al. [27], with the trends for *d*_T-O_ and *T* (Figure 1a) and the trends for *u* and *T* (Figure 1b) overlapping within uncertainty. The trends for *d*_M-O_ and *T* show some difference (Figure 1c), which can be mostly attributed to the small amounts of Fe, Cr, and Ti impurities in our MgAl_2_O_4_-spinel samples. These impurity cations are larger than Al^3+^ [33,47] and appear on the M-sites so that they slightly enlarge the M-sites. The trends for *a* and *T* show even larger difference (Figure 1d). This is simply because *a* is strongly dependent on *d*_M-O_ [26,48],

(8) $$a=\frac{8}{11\sqrt{3}}\left[5d_{T-O}+\sqrt{33{\left(d_{M-O}\right)}^{2}-8{\left(d_{T-O}\right)}^{2}}\right]$$

Nevertheless, the trends defined by our results and those from Andreozzi et al. [27] show some differences in their slopes (Figure 1). The reason is presently unclear.

**Figure 2:**
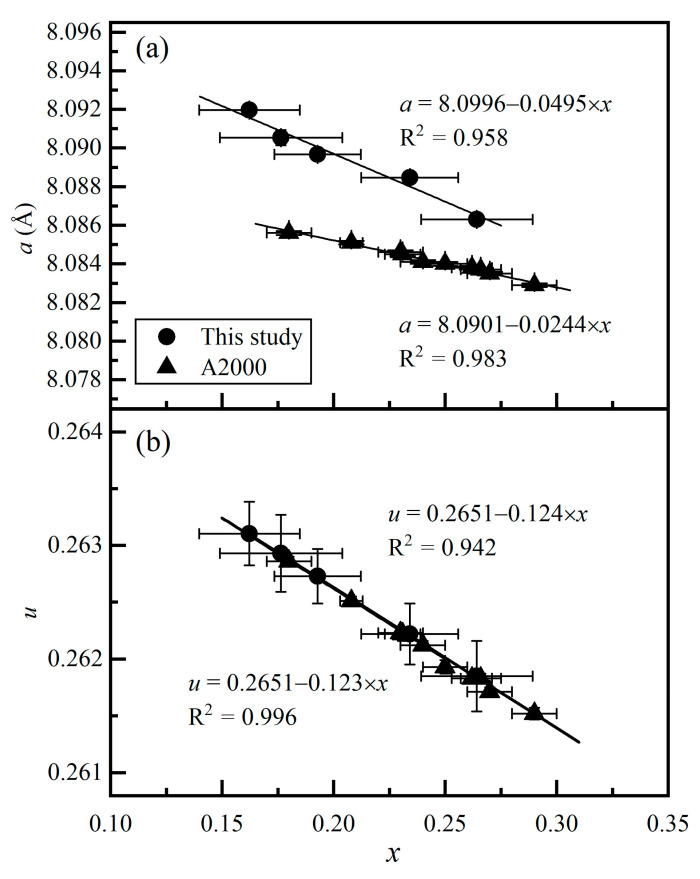
Correlations between *x* and crystal structure parameters of MgAl_2_O_4_-spinel: (**a**) *a* vs. *x*; (**b**) *u* vs. *x*. A2000, Andreozzi et al. [27]. Note that synthetic stoichiometric MgAl_2_O_4_-spinels were used in Andreozzi et al. [27].

Figure 2 shows the relationships between *a* and *x*, and between *u* and *x*. In both cases, good linear correlation is observed for our data. Furthermore, Figure 2a shows that the trends for *a* and *x* defined by the results from this study and by those from Andreozzi et al. [27] have some difference, which can be similarly attributed to the small amounts of Fe, Cr, and Ti impurities in our MgAl_2_O_4_-spinel samples. In contrast, Figure 2b shows that the trends for *u* and *x* defined by the results from this study and by those from Andreozzi et al. [27] are almost identical. This is significant. The *u* parameter is both dependent on *d*_T-O_ and *d*_M-O_ [26,48],

(9) $$u=\frac{0.75\times \frac{{\left(d_{M-O\;}\right)}^{2}}{{\left(d_{T-O}\right)}^{2}}-2+\sqrt{\frac{33{\left(d_{M-O}\right)}^{2}}{16{\left(d_{T-O}\right)}^{2}}-0.5}}{6\left(\frac{{\left(d_{M-O}\right)}^{2}}{{\left(d_{T-O}\right)}^{2}}-1\right)},$$

and captures the essentials of the structural distortion caused by the cation exchange reaction in the spinel structure. The excellent agreement shown for *u* and *x* in Figure 2b suggests that the small amounts of Fe, Cr, and Ti impurities in our MgAl_2_O_4_-spinel samples by no means significantly alter the Mg–Al cation exchange reaction in MgAl_2_O_4_-spinel, supporting the statement made by Ma and Liu [43].

**Figure 3:**
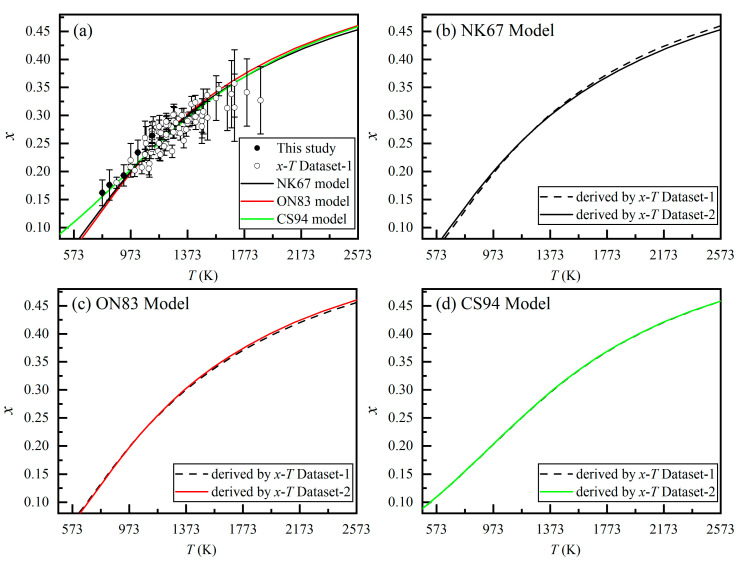
(**a**) New *x*-*T* data from this study and previous reliable *x*-*T* data assembled by Ma and Liu [43] (*x*-*T* Dataset 1), along with the NK67 Model, ON83 Model, and CS94 Model constructed by using the *x*-*T* Dataset 2; (**b**) the NK67 Model derived by using the *x*-*T* Dataset 1 and Dataset 2; (**c**) the ON83. Model thermodynamic model derived by using the *x*-*T* Dataset 1 and Dataset 2; (**d**) the CS94 Model derived by using the *x*-*T* Dataset 1 and Dataset 2.

Our *x*-*T* data (0.162(23) ≤ *x* ≤ 0.258(25) and 773 ≤ *T* ≤ 1123 K) are summarized in Figure 3a, along with those 71 pairs of equilibrium *x*-*T* data selected by Ma and Liu [43] from previous experimental studies (873 ≤ *T* ≤ 1887 K and 0.18(1) ≤ *x* ≤ 0.357(60)). These data are deemed as generally reliable. For convenience, we hereafter refer to the 71 pairs of data from Ma and Liu [43] as Dataset 1 and refer our new data (5 pairs) plus the data in Dataset 1 as Dataset 2 (76 pairs of data).

As shown in Figure 3a, the *x*-*T* data range has been just slightly extended by our experiments, by 100 K and ~0.02 in terms of *T* and *x*, respectively. This small data range extension, however, is very important. It clearly discriminates the NK67 Model, the ON83 Model, and the CS94 Model established by Ma and Liu [43], with the CS94 Model much better describing the experimental data at low *T*.

Fitting the *x*-*T* data in Dataset 2 with Equations (4)–(6), we have derived new parameters for the NK67 Model, the ON83 Model, and the CS94 Model, with the parameters as Δ*H*_D,int_ = 29.22(19) kJ·mol^−1^ (R^2^ = 0.851), α = 27.99(128) kJ·mol^−1^ and β = 2.48(247) kJ·mol^−1^ (R^2^ = 0.851), and *T*_C_ = 2.1(1662) K and c′ = 1.003(596) (*R*^2^ = 0.937), respectively. As to the NK67 Model (Figure 3b), the old version from Ma and Liu [43] (Δ*H*_D,int_ = 29.30(19) kJ·mol^−1^) underestimates the *x* values at *T* < ~973 K but overestimates the *x* values at *T* > ~1473 K. The old version of the ON83 Model from Ma and Liu [43] (*α* = 28.63(136) kJ·mol^−1^ and β = 1.35(261) kJ·mol^−1^) performs slightly better but overestimates the *x* values at *T* < ~673 K and underestimates the *x* values at *T* > ~ 1473 K to small amounts (Figure 3c). In contrast, the old version of the CS94 Model from Ma and Liu [43] (*T*_C_ = 2.2(1825) K and c*′* = 1.00(65)) describes the data equally as well as our new version does. We thus believe that the CS94 Model better describes the Mg–Al cation exchange reaction in the MgAl_2_O_4_-spinel for a wide *T* range at 1 atm.

These three models are all based on Equation (3), but they have different assumptions. For example, the CS94 Model takes into account the Δ*S*_D_ item in Equation (3) [35,49], but the other two models do not. Ignoring the Δ*S*_D_ item is likely inappropriate, considering the obviously smaller residual entropy determined in some calorimetric measurements than the disorder entropy calculated using a random mixing model [22,38]. According to a recent evaluation [43], the Δ*S*_D_ and Δ*S*_C_ items might be of the same order of magnitudes. In addition, the CS94 Model assumes *Q* = 1 at *T* = 0 K (i.e., *x* = 0 at *T* = 0 K), which is embedded in the model-fitting process [36]. *Q* = 1 at *T* = 0 K, i.e., no Mg–Al cation disorder at absolute zero temperature, seems reasonable.

The thermodynamic models established for the cation exchange reaction between the T-sites and M-sites in the spinel structure can be used as a geothermometer to estimate the closure temperature (*T*_-closure_) of the cation exchange process and are thus useful in tracing the thermal history of the spinel-bearing geological bodies. Della Giusta et al. [50] and Princivalle et al. [51] made some pioneering contributions in this field. The spinels they investigated were some Mg–Al rich spinels with certain amounts of Fe^2+^ (up to 0.238 per formula unit), Fe^3+^ (up to 0.059 per formula unit), Cr^3+^, and Ni^2+^. Such high levels of additional cations may bring important influences on the Mg–Al cation exchange equilibrium and highly possibly influence the *T*_-closure_ estimates. It is thus suggested that such a thermometer be applied to spinels with similar compositions.

**Figure 4:**
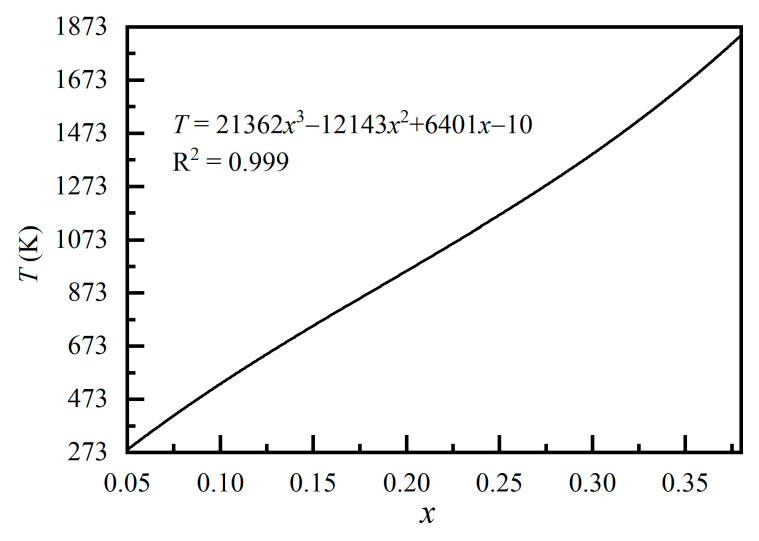
Geothermometer: closure temperature (*T*_-closure_) vs. Mg–Al cation order-disorder (on the basis of the CS94 Model).

Nearly pure MgAl_2_O_4_-spinel has been frequently observed in the fields [20,21,22,23,24,25,52,53,54,55], so there is a need for a geothermometer to estimate the closure temperatures of their cation exchange process. Here we have made such a calibration based on the CS94 Model (Figure 4). The accuracy in the *T*_-closure_ estimates by using this geothermometer is mostly about ±10 K.

## 4. Conclusions

Prolonged annealing experiments have been performed at 773–1123 K and 1 atm to extend the *x*-*T* data range for the Mg–Al cation exchange reaction in MgAl_2_O_4_-spinel. To ensure close equilibrium in these experiments, the heating durations were set to at least twice those inferred from existing kinetics study. The experimental products were analyzed by using single-crystal X-ray diffraction analyses, yielding *x* values ranging from 0.162(23) to 0.258(25) as *T* increased from 773 to 1123 K.

Our new *x*-*T* data were combined with those relatively reliable *x*-*T* data assembled by Ma and Liu [43] to derive new parameters for three thermodynamic models. For the NK67 Model, the new Δ*H*_D,int_ value is 29.22(19) kJ·mol^−1^; for the ON83 Model, the new *α* value is 27.99(128) kJ·mol^−1^, and the new *β* value is 2.48(247) kJ·mol^−1^; for the CS94 Model, the new *T*_C_ value is 2.1(1662) K, and the new *c*′ value is 1.003(596). It was found that the CS94 model describes the experimental data better. This observation might be further examined at high temperatures (e.g., *T* > 1500 K) using some novel experimental techniques, such as the pulsed laser annealing technique with superfast *T*-quenching capability [56,57].

On the basis of the CS94 Model, a geothermometer has been constructed that can be used to estimate the closure temperature (*T*_-closure_) for the Mg–Al cation exchange process in MgAl_2_O_4_-spinel and thereby to infer the thermal history of the geological bodies containing MgAl_2_O_4_-spinel: *T*_-closure_ = 21,362 × *x*^3^ − 12,143 × *x*^2^ + 6401 × *x* − 10. The accuracy of this geothermometer is probably around ±10 K.

## Figures and Tables

**Table 1:** Key parameters of annealing experiments and results of single-crystal X-ray diffraction analyses.

Run #	HT3-1	HT3-2	HT3-3	HT3-4	HT3-5
***T*** (**K**)	1123	1023	923	823	773
***x*** **_-int_** ^1^	~0.145	~0.24	~0.24	~0.24	~0.18
***x*** **_-cal_** ^2^	~0.24	~0.22	~0.20	~0.18	~0.17
***t*** **_-cal_** ^3^	~11.9 h	~1.88 h	~1.32 d	~37.1 d	~147 d
***t*** **_-exp_** ^4^	24 h	7 d	30 d	120 d	360 d
***a***(**Å**)	8.0863(1) ^5^	8.0885(1)	8.0897(2)	8.0905(4)	8.0920(3)
***u***	0.2619(3)	0.2622(3)	0.2627(2)	0.2629(3)	0.2631(3)
***d*_T-O_**(**Å**)	1.91666(1)	1.92239(2)	1.92982(3)	1.93284(6)	1.93562(5)
***d*_M-O_**(**Å**)	1.93054(3)	1.92835(4)	1.92496(5)	1.92372(6)	1.92281(8)
***x***	0.258(25)	0.227(22)	0.190(19)	0.173(27)	0.162(23)
**U_eq_**(**T**) (**Å^2^**)	0.0008(2)	0.0006(1)	0.0004(1)	0.0002(1)	0.0001(1)
**U_eq_**(**M**) (**Å^2^**)	0.0008(2)	0.0006(1)	0.0004(1)	0.0002(1)	0.0001(1)
**U_eq_**(**O**) (**Å^2^**)	0.0008(2)	0.0007(1)	0.0008(1)	0.0007(1)	0.0005(1)
**Refl.**	47	47	59	58	60
***R*_int_**(**%**)	9.10	4.79	4.29	4.67	4.12
***R*_1_**(**%**)	2.07	2.06	3.36	4.04	3.89
**w*R*_2_** (**%**)	9.03	8.96	8.46	9.76	8.73
**GooF**	1.004	1.046	1.006	1.066	1.013

^1^ The initial *x* value of the MgAl_2_O_4_-spinel sample for certain annealing experiments. ^2^ The expected equilibrium *x* value of the MgAl_2_O_4_-spinel sample at certain *T*, as calculated by using the ON83 Model in Ma and Liu [43]. With the only exception of HT3-1, the equilibrium state in all other experiments was approached by the Mg–Al cation ordering process (i.e., *x* decreasing), which has a much faster reaction rate than the Mg–Al cation disordering process [42,43]. ^3^ The expected heating duration for the MgAl_2_O_4_-spinel sample at certain *T* to reach its cation exchange equilibrium, as calculated by using Equation (8) in Andreozzi and Princivalle [42]. ^4^ The real experimental annealing duration, which had been set significantly longer than the expected heating duration (*t*_-cal_). ^5^ Number in the parentheses is one standard deviation in the rightmost digit.

## Data Availability

All supporting data can be found in the paper and its Supplementary Materials.

## Supplementary Materials

The following are available online, Tables S1–S5: Details of structure refinement of spinel sample of run HT3-1 to HT3-5) and Cif files Cif-HT3-1 to Cif-HT3-5.

## Sample Availability

Samples of the compounds are available from the authors.
