# Supplementary material for: Thermodynamics of Mg–Al Order-Disorder Reaction in MgAl2O4-Spinel: Constrained by Prolonged Annealing Experiments at 773–1123 K
Source: Molecules. 2021 Feb 7;26(4):872. doi: 10.3390/molecules26040872 (PMC7914506; doi:10.3390/molecules26040872)
Supplement: Supplementary file 1 [file molecules-26-00872-s001.zip › SupplementaryMaterial/SupplementaryMaterials.pdf]

Supplementary Material for

Thermodynamics of Mg-Al Order-Disorder Reaction in $MgAl_2O_4$-Spinel: Constrained by Prolonged Annealing Experiments at 773–1123 K

Yunlu Ma [1,2], Xinjian Bao [1,2] and Xi Liu [1,2,*]

[1] School of Earth and Space Sciences, Peking University, Beijing 100871, China; Yunlu.Ma@pku.edu.cn
[2] Key Laboratory of Orogenic Belts and Crustal Evolution, Ministry of Education of China, Beijing 100871, China; Xinjian.Bao@pku.edu.cn
* Correspondence: Xi.Liu@pku.edu.cn; Tel.: +86-10-6275-3585; Fax: +86-10-6275-2996

List of Supplementary Material Tables and Files

STABLE. 1 Details of structure refinement of spinel sample of run HT3-1
STABLE. 2 Details of structure refinement of spinel sample of run HT3-2
STABLE. 3 Details of structure refinement of spinel sample of run HT3-3
STABLE. 4 Details of structure refinement of spinel sample of run HT3-4
STABLE. 5 Details of structure refinement of spinel sample of run HT3-5
Cif file 1: Cif HT3-1 (for $MgAl_2O_4$-spinel sample HT3-1)
Cif file 2: Cif HT3-2 (for $MgAl_2O_4$-spinel sample HT3-2)
Cif file 3: Cif HT3-3 (for $MgAl_2O_4$-spinel sample HT3-3)
Cif file 4: Cif HT3-4 (for $MgAl_2O_4$-spinel sample HT3-4)
Cif file 5: Cif HT3-5 (for $MgAl_2O_4$-spinel sample HT3-5)

STABLE. 1 Details of structure refinement of spinel sample of run HT3-1

Chemical formula sum	$Mg_{1.000(3)}Al_{1.977(3)}Cr_{0.011(1)}Fe_{0.006(1)}Ti_{0.002(1)}O_4$	
Formula weight	142.699	
Temperature	293(2) K	
Wavelength	0.71073 Å	
Crystal system	Cubic	
Space group	$Fd\bar{3}m$	
Unit cell dimensions	a = 8.0863(1) Å	α = 90 º
	b = 8.0863(1) Å	β = 90 º
	c = 8.0863(1) Å	γ = 90 º
Volume	528.75(2) $Å^3$	
Z	8	
Theta range for data collection	4.37 to 28.25º	
Index ranges	$-10 \le h \le 10$, $-10 \le k \le 10$, $-9 \le l \le 10$	
Reflections collected	1082	
Independent reflections	47 [R(int) = 0.0910]	
Completeness to theta = 28.25 º	100.0 %	
Refinement method	Full-matrix least-squares on F^2	
Data / restraints / parameters	47 / 0 / 8	
Goodness-of-fit on F^2	1.004	
Final R indices [I>2sigma(I)]	R_1 = 0.0270, wR_2 = 0.0903	
R indices (all data)	R_1 = 0.0270, wR_2 = 0.0903	
Largest diff. peak and hole	0.290 and -0.698 e.$Å^{-3}$	

STABLE. 2 Details of structure refinement of spinel sample of run HT3-2

Chemical formula sum	$Mg_{1.000(3)}Al_{1.977(3)}Cr_{0.011(1)}Fe_{0.006(1)}Ti_{0.002(1)}O_4$	
Formula weight	142.699	
Temperature	293(2) K	
Wavelength	0.71073 Å	
Crystal system	Cubic	
Space group	$Fd\bar{3}m$	
Unit cell dimensions	a = 8.0885(1) Å	α = 90 °
	b = 8.0885 (1) Å	β = 90 °
	c = 8.0885(1) Å	γ = 90 °
Volume	529.17(2) $Å^3$	
Z	8	
Theta range for data collection	4.37 to 28.24°	
Index ranges	$-10 \le h \le 10$, $-10 \le k \le 10$, $-9 \le l \le 10$	
Reflections collected	1071	
Independent reflections	47 [R(int) = 0.0479]	
Completeness to theta = 28.25 °	100.0 %	
Refinement method	Full-matrix least-squares on F^2	
Data / restraints / parameters	47 / 0 / 8	
Goodness-of-fit on F^2	1.046	
Final R indices [I>2sigma(I)]	R_1 = 0.0206, wR_2 = 0.0896	
R indices (all data)	R_1 = 0.0206, wR_2 = 0.0896	
Largest diff. peak and hole	0.286 and -0.295 e.$Å^{-3}$	

STABLE. 3 Details of structure refinement of spinel sample of run HT3-3

Chemical formula sum	$Mg_{1.000(3)}Al_{1.977(3)}Cr_{0.011(1)}Fe_{0.006(1)}Ti_{0.002(1)}O_4$	
Formula weight	142.699	
Temperature	293(2) K	
Wavelength	0.71073 Å	
Crystal system	Cubic	
Space group	$Fd\bar{3}m$	
Unit cell dimensions	a = 8.0897(2) Å	α = 90 º
	b = 8.0897(2) Å	β = 90 º
	c = 8.0897(2) Å	γ = 90 º
Volume	529.41(2) $Å^3$	
Z	8	
Theta range for data collection	4.36 to 31.32º	
Index ranges	$-10 \leq h \leq 10$, $-10 \leq k \leq 10$, $-9 \leq l \leq 10$	
Reflections collected	1071	
Independent reflections	59 [R(int) = 0.0429]	
Completeness to theta = 31.32 º	100.0 %	
Refinement method	Full-matrix least-squares on F^2	
Data / restraints / parameters	59 / 0 / 7	
Goodness-of-fit on F^2	1.006	
Final R indices [I>2sigma(I)]	R_1 = 0.0336, wR_2 = 0.0846	
R indices (all data)	R_1 = 0.0336, wR_2 = 0.0846	
Largest diff. peak and hole	0.377 and -0.778 e.$Å^{-3}$	

STABLE. 4 Details of structure refinement of spinel sample of run HT3-4

Chemical formula sum	$Mg_{1.000(3)}Al_{1.977(3)}Cr_{0.011(1)}Fe_{0.006(1)}Ti_{0.002(1)}O_4$	
Formula weight	142.699	
Temperature	293(2) K	
Wavelength	0.71073 Å	
Crystal system	Cubic	
Space group	$Fd\bar{3}m$	
Unit cell dimensions	a = 8.0905(4) Å	α = 90 º
	b = 8.0905(4) Å	β = 90 º
	c = 8.0905(4) Å	γ = 90 º
Volume	529.58(5) $Å^3$	
Z	8	
Theta range for data collection	4.36 to 31.19º	
Index ranges	$-10 \le h \le 10$, $-10 \le k \le 10$, $-9 \le l \le 10$	
Reflections collected	1071	
Independent reflections	58 [R(int) = 0.0467]	
Completeness to theta = 31.19 º	100.0 %	
Refinement method	Full-matrix least-squares on F^2	
Data / restraints / parameters	58 / 0 / 7	
Goodness-of-fit on F^2	1.066	
Final R indices [I>2sigma(I)]	R_1 = 0.0404, wR_2 = 0.0976	
R indices (all data)	R_1 = 0.0407, wR_2 = 0.0976	
Largest diff. peak and hole	0.597 and -0.778 e.$Å^{-3}$	

STABLE. 5 Details of structure refinement of spinel sample of run HT3-5

Chemical formula sum	$Mg_{1.000(3)}Al_{1.977(3)}Cr_{0.011(1)}Fe_{0.006(1)}Ti_{0.002(1)}O_4$	
Formula weight	142.699	
Temperature	293(2) K	
Wavelength	0.71073 Å	
Crystal system	Cubic	
Space group	$Fd\bar{3}m$	
Unit cell dimensions	a = 8.0920(3) Å	α = 90 °
	b = 8.0920(3) Å	β = 90 °
	c = 8.0920(3) Å	γ = 90 °
Volume	529.86(4) $Å^3$	
Z	8	
Theta range for data collection	4.36 to 31.18°	
Index ranges	$-10 \le h \le 10$, $-10 \le k \le 10$, $-9 \le l \le 10$	
Reflections collected	1071	
Independent reflections	60 [R(int) = 0.0412]	
Completeness to theta = 31.18 °	100.0 %	
Refinement method	Full-matrix least-squares on F^2	
Data / restraints / parameters	60 / 0 / 7	
Goodness-of-fit on F^2	1.013	
Final R indices [I>2sigma(I)]	R_1 = 0.0389, wR_2 = 0.0873	
R indices (all data)	R_1 = 0.0389, wR_2 = 0.0873	
Largest diff. peak and hole	0.991 and -1.034 e.$Å^{-3}$	
